# Does Density Ratio Significantly Affect Turbulent Flame Speed?

**DOI:** 10.1007/s10494-017-9801-6

**Published:** 2017-02-15

**Authors:** A. N. Lipatnikov, W. Y. Li, L. J. Jiang, S. S. Shy

**Affiliations:** 1 Department of Applied Mechanics, Chalmers University of Technology, Gothenburg, Sweden; 2 Department of Mechanical Engineering, National Central University, Jhong-li, Taiwan

**Keywords:** Turbulent flame speed, Density ratio, Expanding spherical flame, Thermal expansion, Low Turbulent Intensity, Darrieus-Landau instability, Experiment

## Abstract

In order to experimentally study whether or not the density ratio *σ* substantially affects flame displacement speed at low and moderate turbulent intensities, two stoichiometric methane/oxygen/nitrogen mixtures characterized by the same laminar flame speed *S*_*L*_ = 0.36 m/s, but substantially different *σ* were designed using (i) preheating from *T*_*u*_ = 298 to 423 K in order to increase *S*_*L*_, but to decrease *σ*, and (ii) dilution with nitrogen in order to further decrease *σ* and to reduce *S*_*L*_ back to the initial value. As a result, the density ratio was reduced from 7.52 to 4.95. In both reference and preheated/diluted cases, direct images of statistically spherical laminar and turbulent flames that expanded after spark ignition in the center of a large 3D cruciform burner were recorded and processed in order to evaluate the mean flame radius $\bar {R}_{f}\left (t \right )$ and flame displacement speed $S_{t}=\sigma ^{-1}{d\bar {R}_{f}} \left / \right . {dt}$ with respect to unburned gas. The use of two counter-rotating fans and perforated plates for near-isotropic turbulence generation allowed us to vary the rms turbulent velocity $u^{\prime }$ by changing the fan frequency. In this study, $u^{\prime }$ was varied from 0.14 to 1.39 m/s. For each set of initial conditions (two different mixture compositions, two different temperatures *T*_*u*_, and six different $u^{\prime })$, five (respectively, three) statistically equivalent runs were performed in turbulent (respectively, laminar) environment. The obtained experimental data do not show any significant effect of the density ratio on *S*_*t*_. Moreover, the flame displacement speeds measured at *u*′/*S*_*L*_ = 0.4 are close to the laminar flame speeds in all investigated cases. These results imply, in particular, a minor effect of the density ratio on flame displacement speed in spark ignition engines and support simulations of the engine combustion using models that (i) do not allow for effects of the density ratio on *S*_*t*_ and (ii) have been validated against experimental data obtained under the room conditions, i.e. at higher *σ*.

## Introduction

As reviewed elsewhere [1–4], turbulent flame speed *S*_*t*_ was in the focus of experimental research into premixed turbulent combustion for decades, with a number of new experimental data bases being built over the past twelve years, e.g. see [5–18]. Results of such measurements are often reported in a form of a parameterization of *S*_*t*_ as a function of the rms magnitude $u^{\prime }$ of turbulent velocity fluctuations, an integral length scale *L* of turbulence, the laminar flame speed *S*_*L*_ and thickness *δ*_*L*_, and eventually Lewis *L* *e* or Markstein *M* *a* number. However, to the best of the present authors’ knowledge, such parameterizations do not involve a ratio $\sigma =\rho _{u} \left / \right . \rho _{b}$ of the densities of unburned and burned mixtures, thus, implying a minor effect of *σ* on *S*_*t*_.

On the contrary, as reviewed elsewhere [19–21], there are a number of well-documented phenomena associated with the influence of combustion-induced density variations on turbulent flow and transport within a premixed flame brush, with these phenomena being argued by many experts to substantially affect *S*_*t*_. For instance, turbulent flame speed was hypothesized to be affected by

- flame-generated turbulence highlighted by Karlovitz et al. [22] and Scurlock and Grover [23] and investigated in a number of subsequent experimental papers reviewed elsewhere [19–21], e.g. see Direct Numerical Simulation (DNS) study by Poludnenko [24] as a recent example,
- counter-gradient transport (criteria of its appearance [25–27] and various simple models [28–32] consider *σ* to be an important input parameter, with the influence of the magnitude of the counter-gradient flux on *S*_*t*_ being theoretically proved [33, 34]),
- the local Darrieus-Landau (DL) instability [35] of thin, inherently laminar flame fronts (flamelets), caused by the local density drop (some of models that address influence of the DL instability on premixed turbulent combustion straightforwardly yield an increase in *S*_*t*_ by *σ* [36, 37], whereas other models yield an increase in *S*_*t*_ with decreasing *M* *a* [38] or a neutral wavelength^1^ *λ*_*n*_ of the DL instability [40]),
- variations in the mean scalar dissipation rate $\bar {\varepsilon }_{c}= \overline {D \nabla c \cdot \nabla c}$ due to dilatation [41], where *D* is the molecular diffusivity and *c* is the combustion progress variable.

The reader interested in a deeper discussion of these phenomena and models is referred to recent review papers [20, 21, 42]. Here, we restrict ourselves to pointing out that a substantial effect of the density ratio on turbulent flame speed is widely expected in theoretical and numerical combustion community, but such an effect is not indicated by experimental parameterizations of *S*_*t*_.

At first glance, this apparent contradiction challenges the aforementioned concepts and models. However, the following counterargument may be put forward in order to defend them. Because variations in mixture composition are accompanied by simultaneous variations in *S*_*L*_, *δ*_*L*_, and *σ*, separation of the effect of any of these quantities on *S*_*t*_ from the effects of other two quantities is difficult. Accordingly, when analyzing measured data, researchers often (i) highlight *S*_*L*_ to be the primary mixture characteristic that controls turbulent combustion rate and, sometimes, (ii) allow for dependence of *S*_*t*_ also on *δ*_*L*_, thus, leaving eventual effect of *σ* on *S*_*t*_ beyond the scope of a typical experimental study. Consequently, in the available parameterizations of experimental data on *S*_*t*_, eventual density-ratio effects may be hidden in dependencies of *S*_*t*_ on *S*_*L*_ and *δ*_*L*_. Therefore, in order to either challenge or support models that yield a substantial dependence of *S*_*t*_ on *σ*, e.g. [22, 23, 36, 37, 41], a target-directed experimental research into eventual influence of *σ* on *S*_*t*_ is required. Wide spread of the aforementioned models calls strongly for such an experimental study.

However, a target-directed experimental investigation of the issue (whether or not *σ* substantially affects *S*_*t*_) is difficult, because variations in fuel formula, equivalence ratio Φ, unburned gas temperature *T*_*u*_, or pressure *P*, performed in the majority of measurements of *S*_*t*_, did not allow researchers to separate the effects of *S*_*L*_ on *S*_*t*_ from eventual influence of *σ* on *S*_*t*_, with the former effects being definitely significant. The present authors are aware on a single attempt to address the issue by substantially changing the density ratio in experiments. To do so, Burluka et al. [43] measured speeds of flames of di-t-butyl-peroxide (DTBP) decomposition in a 0.376DTBP + 1.0N2 mixture in the well-known Leeds fan-stirred bomb. In spite of a low value of *σ* = 3.57, the documented dependencies of *S*_*t*_ on $u^{\prime }$ were “in good agreement” with experimental data obtained by other research groups from hydrocarbon-air mixtures “with similar laminar flame speed and Lewis number” [43], thus, implying a minor effect of *σ* on *S*_*t*_. However, such a conclusion appears to be insufficiently solid, because it was drawn by comparing the Leeds DTBP data with data obtained using other techniques in other laboratories.

The discussed issue can straightforwardly be studied in a DNS, where variations in *σ* can be performed by retaining the same values of *S*_*L*_, *δ*_*L*_, etc. However, available data are controversial. For instance, on the one hand, Treurniet et al. [44] simulated propagation of an infinitely thin front in 3D turbulence and reported an increase in $S_{t} \left / \right . S_{L}$ by *σ*. On the other hand, Fig. 1 from [45] does not indicate a substantial effect of *σ* on the mean (time-averaged) $\overline {S_t(t)/S_L}$ in the flamelet regime of premixed turbulent combustion. Recent unsteady 2D simulations of hydrodynamically unstable (“supercritical”) flames [46, 47] indicate a weak dependence of *S*_*t*_ on *σ* in spite of substantial influence of the DL instability on the simulated flames.

In summary, first, there are a number of well-documented phenomena associated with significant effects of density variations on turbulent flow and transport in premixed flames and, in particular, a number of concepts and models that yield an increase in *S*_*t*_ by *σ*. However, second, the present authors are not aware on experimental data that clearly show an increase in *S*_*t*_ by *σ*. However, third, the lack of such data may be attributed to limited capabilities of a typical experimental study to separate the influence of the density ratio on turbulent flame speed from the influence of *S*_*L*_ and *δ*_*L*_ on *S*_*t*_. Thus, to the best of the present authors’ knowledge, it is not yet clear whether or not *σ* affects *S*_*t*_ substantially. This fundamental issue is still waiting for a target-directed experimental investigation and appears to be of great importance for development and assessment of advanced models of premixed turbulent combustion. Accordingly, the goal of the present work is to contribute to filling this knowledge gap by measuring turbulent flame displacement speeds in mixtures that are specially prepared by combining preheating and dilution of unburned reactants in order to substantially change *σ*, but retain *S*_*L*_ unchanged. Such experimental arrangements and conditions are described in the next section. Obtained results are reported in Section 3 and are discussed in Section 4, followed by conclusions.

Finally, it is worth stressing that, because the aforementioned models and concepts predict the strongest effect of thermal expansion on turbulent flow and, hence, on *S*_*t*_ under conditions of weak turbulence (${u^{\prime }} \left / \right . S_{L}<1)$, as reviewed elsewhere [20, 21], the focus of the present experimental study is placed on such conditions.

## Experimental Conditions and Arrangements

### Method of research

When a combustible mixture is preheated, its laminar flame speed is increased, but the density ratio is decreased. When the mixture is diluted with nitrogen, both *S*_*L*_ and *σ* are decreased. Accordingly, combination of these two options allows us (i) to retain *S*_*L*_ unchanged by counterbalancing an increase in *S*_*L*_ due to preheating with a decrease in *S*_*L*_ due to dilution, but (ii) to substantially change *σ*, which is reduced both by the dilution and preheating.

In the present work, this idea is applied to the stoichiometric CH_4_/O_2_/N_2_ mixtures. First, we measured dependence of a turbulent flame displacement speed *S*_*t*_ on $u^{\prime }$ for the stoichiometric methane-air mixture under the room conditions. Second, dependence of $S_{t}\left (u^{\prime } \right )$ was also measured for the same mixture, which was uniformly preheated to *T*_*u*_ = 423 K. Third, the preheated mixture was diluted with nitrogen in order to reduce *S*_*L*_ back to the reference value associated with the room conditions. The required amount of nitrogen was preliminarily estimated using the GRI chemical mechanism [48] and running PREMIX code [49]. Subsequently, the concentration of nitrogen in the diluted mixture was experimentally adjusted based on the measured *S*_*L*_ (at *T*_*u*_ = 423 K). It is worth noting that the use of the stoichiometric CH_4_/O_2_/N_2_ mixtures allows us to reduce preferential diffusion and Lewis number effects, which are discussed in detail elsewhere [50].

Characteristics of three investigated mixtures are reported in Table 1. The volume percentage of N_2_ was calculated in the diluted mixture. The density ratios were evaluated by computing composition and temperature of adiabatic equilibrium combustion products, with the same results being obtained using an in-house code and CHEMKIN [51] software. The laminar flame thicknesses $\delta _{L}=\left (T_{b}-T_{u} \right ) \left / \right . {\max \left | \mathrm {\nabla } T \right |}$ were computed using the GRI chemical mechanism [48] and running PREMIX code [49]. The laminar flame speeds *S*_*L*_ were determined by processing measured $R_{f}\left (t \right )$ data using four methods discussed elsewhere [52]. Because the investigated mixtures are characterized by *L* *e*≈1 and small Markstein lengths, all these methods yielded approximately the same *S*_*L*_ and the values reported in Table 1 have been obtained by considering the difference in the observed flame speed ${dR_{f}} \left / \right . {dt}$ and *σ* *S*_*L*_ to be a linear function of the flame stretch rate $\dot {s}=\left (2 \left / \right . R_{f} \right ){dR_{f}} \left / \right . {dt}$, i.e. [53]

1 $$\begin{array}{@{}rcl@{}} \frac{dR_{f}}{dt}= \sigma \left( S_{L}-\mathcal{L}_{b}^{exp}\dot{s} \right)=\sigma S_{L}-\sigma \mathcal{L}_{b}^{exp}\dot{s}=\sigma S_{L}-\sigma \mathcal{L}_{b}^{exp}\frac{2}{R_{f}}\frac{dR_{f}}{dt} \end{array} $$

**Table 1:** Mixture characteristics

Case	Diluent	*T*_*u*_	*S*_*L*_,	*δ*_*L*_	$D_{u} \left / \right . S_{L}$	*σ*	$\mathcal {L}_{u}$	$\mathcal {L}_{b}$	$\mathcal {L}_{b}^{exp}$	$\sigma \mathcal {L}_{b}$	$\sigma \mathcal {L}_{b}^{exp}$
	(vol. %)	(K)	(m/s)	(mm)	(mm)		(mm)	(mm)	(mm)	(mm)	(mm)
1	0	423	0.66	0.38	0.055	5.45	0.22	0.040	0.16	0.22	0.87
2	0	298	0.36	0.44	0.056	7.52	0.28	0.038	0.11	0.29	0.83
3	17.17	423	0.36	0.56	0.10	4.95	0.37	0.075	0.21	0.37	1.04

In addition to values of *S*_*L*_, $\mathcal {L}_{b}^{exp}$, and $\sigma \mathcal {L}_{b}^{exp}$, evaluated by applying Eq. 1 to processing experimental data obtained from laminar flames, Table 1 also reports Markstein lengths $\mathcal {L}_{u}$, $\mathcal {L}_{b}$, and $\sigma \mathcal {L}_{b}$ that have been calculated using the following theoretical expressions [53]

2 $$ \mathcal{L}_{u}=\frac{D_{u}}{S_{L}}\frac{\sigma} {\sigma -1}\int\limits_{1}^{\sigma} {\frac{\lambda \left( x \right)}{x}dx} ,\qquad\quad \mathcal{L}_{b}=\mathcal{L}_{u}-\frac{D_{u}}{S_{L}}\int\limits_{1}^{\sigma} {\frac{\lambda \left( x \right)}{x}dx} $$

by assuming that *L* *e* = 1 in the stoichiometric CH_4_/O_2_/N_2_ mixtures. Here, *D*_*u*_ is the molecular diffusivity of CH_4_ in unburned mixture, and $\lambda \left (x \right )=x^{0.7}$ characterize the temperature-dependence of the molecular diffusivity, i.e. $\lambda ={D\left (T \right )} \left / \right . D\left (T_{u} \right )=\left (T \left / \right . T_{u} \right )^{0.7}$ [53]. The fact that the measured $\mathcal {L}_{b}^{exp}$ is larger than the theoretical *ℒ*_*b*_ is not surprising, because (i) Eq. 2 has been derived in the case of a single reaction and (ii) “the more complex the reaction scheme the larger the values of” $\mathcal {L}_{b}$ [53].

As shown in Table 1, the combination of preheating and dilution allowed us to reduce the density ratio from 7.52 to 4.95, i.e. by 34 %, by retaining the same *S*_*L*_ = 0.36 m/s (cases 2 and 3). Even if such a change in *σ* is moderate, it appears to be sufficient in order to observe an effect of *σ* on *S*_*t*_ provided that such an effect is significant. For instance, according to the seminal work by Karlovitz et al. [22], the rms magnitude of flame-generated velocity is proportional to *τ* = *σ* − 1, which is equal to 6.52 and 3.95 in cases 2 and 3, respectively. Moreover, the normalized growth rate ${\sigma \left (\sqrt {1+\sigma -1 \left / \right . \sigma } -1 \right )} \left / \right . \left (\sigma +1 \right )$ of the DL instability [35] is equal to 1.67 and 1.16 in cases 2 and 3, respectively, thus, implying that *S*_*t*, 2_ should be notably larger than *S*_*t*, 3_ if the instability plays a substantial role under conditions of the present experiments. Henceforth, digital subscripts indicate the case number. In particular, *S*_*t*_ is proportional to this growth rate within the framework of a model developed by Kuznetsov and Sabelnikov [36]. Furthermore, if stabilization of a 2D laminar flame due to nonlinear effects is considered to be relevant, then, dependence of an increase in the flame speed due to the DL instability on the density ratio is controlled by ${\sigma \left (\sigma -1 \right )^{2}} \left / \right . \left (\sigma ^{3}+\sigma ^{2}+3\sigma -1 \right )$ [54], which is equal to 0.64 and 0.48 in cases 2 and 3, respectively, thus, again implying a notable difference in *S*_*t*, 2_ and *S*_*t*, 3_. Finally, according to a model by Kolla et al. [41], $S_{t}\propto \sqrt \tau $ if ${u^{\prime }} \left / \right . S_{L}$ is sufficiently low and $\sqrt \tau \approx 2.6$ and 2.0 in cases 2 and 3, respectively. Thus, there are a number of models that predict a notable decrease in turbulent flame speed when decreasing the density ratio from 7.52 to 4.95.

Table 1 also shows that the combination of preheating and dilution does not allow us to retain the same thickness *δ*_*L*_, which is larger in case 3 than in case 2. To understand whether or not this difference in *δ*_*L*, 2_ and *δ*_*L*, 3_ can cast doubt on results reported in Section 3, let us discuss how variations in *δ*_*L*_ can affect *S*_*t*_.

First, if we consider approximations of the most extensive experimental databases on *S*_*t*_ obtained from expanding statistically spherical flames [3, 4, 55], then, the following three points are of importance for the present discussion. These approximations (i) do not involve the density ratio, (ii) were obtained by ignoring the DL instability, and (iii) yield an increase in $S_{t} \left / \right . S_{L}$ by both ${u^{\prime }} \left / \right . S_{L}$ and $L \left / \right . \delta _{L}$, with the latter trend being much less pronounced, e.g. $S_{t} \left / \right . S_{L}\propto \left ({u^{\prime }} \left / \right . S_{L} \right )^{3 \left / \right . 4}\left (L \left / \right . \delta _{L} \right )^{1 \left / \right . 4}$ [3] or $S_{t} \left / \right . S_{L}\propto \left ({u^{\prime }} \left / \right . S_{L} \right )^{1 \left / \right . 2}\left (L \left / \right . \delta _{L} \right )^{1 \left / \right . 6}$ [4, 55]. Accordingly, variations in $L \left / \right . \delta _{L}$ in a range of 30 % (cf. cases 2 and 3 in Table 1) are not expected to substantially affect *S*_*t*_ under conditions of the present study. Moreover, Table 1 shows that case 3 is characterized by a larger *δ*_*L*_ and a lower *σ* when compared to case 2. Consequently, as argued above, the difference in *δ*_*L*, 2_ and *δ*_*L*, 3_ is expected to slightly increase *S*_*t*, 2_ when compared to *S*_*t*, 3_. Because models of premixed turbulent combustion that predict influence of *σ* on *S*_*t*_ yield ${dS_{t}} \left / \right . {d\sigma } >0$, e.g. [36, 37, 41, 54], the difference in *σ*_2_ and *σ*_3_ is also expected to increase (if any) *S*_*t*, 2_ when compared to *S*_*t*, 3_. Therefore, the differences in (i) *δ*_*L*, 2_ and *δ*_*L*, 3_ and (ii) *σ*_2_ and *σ*_3_ could affect *S*_*t*_ in the same direction, but are unlikely to counteract one another. Because experimental data reported in the next section will show almost equal *S*_*t*, 2_ and *S*_*t*, 3_, this result cannot be attributed to mutual cancellations of the effects of the laminar flame thickness and density ratio on turbulent flame speed.

Second, a change in *δ*_*L*_ straightforwardly affects various Markstein lengths $\mathcal {L}$, which are also affected by the corresponding Markstein numbers, with *M* *a* being mainly controlled by the density ratio if *L* *e* = 1 [39, 53]. Because local variations in a stretched laminar flame speed are proportional to $\left (-\mathcal {L}\dot {s} \right )$ within the framework of the linear theory of laminar flame perturbations [39, 53], an increase in $\mathcal {L}$ is expected to result in decreasing mean local consumption and displacement speeds due to turbulent stretching of flamelets [2, 50, 55]. Moreover, an increase in $\mathcal {L}$ is expected to result in decreasing susceptibility of the flamelets to the DL instability due to an increase in the neutral wavelength *λ*_*n*_ [39, 53]. Both effects are expected to result in decreasing *S*_*t*_. Table 1 shows that two most widely used^2^ Markstein lengths $\mathcal {L}_{b}$ (or $\mathcal {L}_{b}^{exp})$ and $\mathcal {L}_{u}$ are larger in case 3 than in case 2, as well as $\sigma \mathcal {L}_{b}$ or $\sigma \mathcal {L}_{b}^{exp}$. As far as the third most relevant Markstein length *ℒ*_*c*_, which characterizes local consumption speed, is concerned, it vanishes if *L* *e* = 1 within the framework of the linear theory [39, 53]. Therefore, the differences in (i) $\mathcal {L}_{2}$ and $\mathcal {L}_{3}$ and (ii) *σ*_2_ and *σ*_3_ could affect *S*_*t*_ in the same direction, i.e. make *S*_*t*, 2_ larger than *S*_*t*, 3_, but are unlikely to counteract one another. Again, because experimental data reported in the next section will show almost equal *S*_*t*, 2_ and *S*_*t*, 3_, this result cannot be attributed to mutual cancellations of the effects of the Markstein length and density ratio on turbulent flame speed.

### Experimental arrangements

**Fig. 1:**
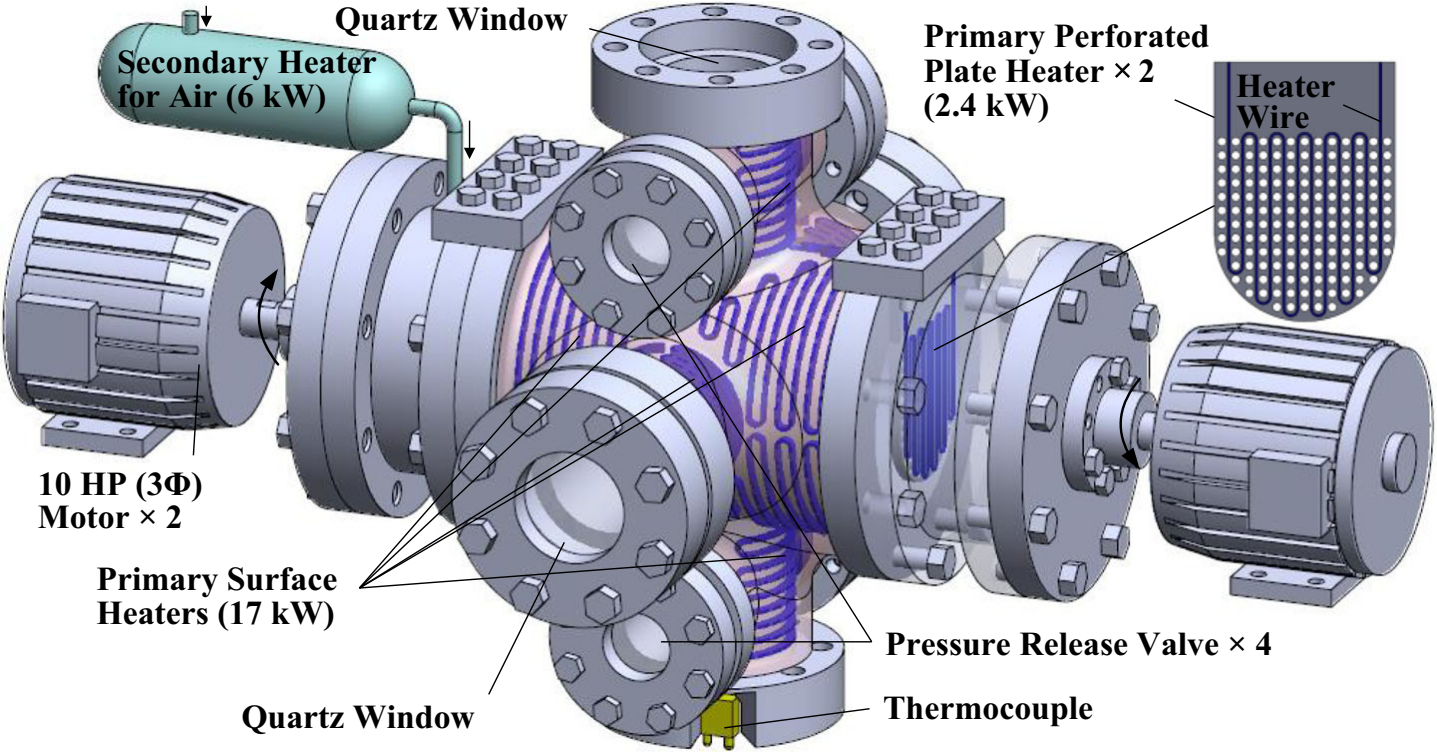
The dual-chamber, fan-stirred 3D cruciform burner with various heating devices resided in a huge pill-like outer vessel (not shown)

Experiments are conducted in a dual-chamber explosion facility that was already used to measure propagation speeds of expanding statistically spherical turbulent flames [13, 56, 57]. The facility consisted of a large inner 3D cruciform burner (see Fig. 1) situated within a huge pill-like outer chamber (not shown). The 3D cruciform burner was constructed by a large horizontally-positioned cylindrical steel pipe together with four smaller cylindrical steel pipes perpendicularly-aligned and symmetrically-welded around its central part to form a cruciform shape when viewed from all three directions. The spherical diameter of the inside intersecting domain from these pipes is about 300 mm.

A pair of counter-rotating fans and perforated plates installed in the burner allow us to create a sizable near-isotropic turbulence flow field, as already discussed in [58–61]. The rms velocity $u^{\prime }$ and an integral length scale *L* of turbulence can simultaneously be varied by changing the fan frequency *f* (Hz). Both LDV and PIV measurements [58–61] have shown that, under conditions of the present experiments, $u^{\prime }=0.0462f$ (m/s) and *L* = 10.7*f* ^0.34^ (mm), with *L* being leveled off to a maximum of 45 mm at high values of *f*. The fact that variations in $u^{\prime }$ are accompanied with significantly weaker variations in *L* is of minor importance for the goal of the present study that aims solely at comparing speeds of the reference and preheated/diluted flames (cases 2 and 3, respectively) that propagate through statistically the same turbulence. It is also worth noting that an increase in *L* by the frequency *f* has not been found in several other fan-stirred combustion bombs [8, 12, 17]. This qualitative difference between the present and other data on the integral length scale *L* is attributed to the use of the two perforated plates in the present experiments. These plates appear to break up the vortical streams generated by the two counter-rotating fans (see Fig. 1) into smaller vortices, while such plates are not installed in other fan-stirred bombs, to the best of the present authors’ knowledge.

For the goal of the present work, we applied the newly-modified cruciform burner equipped with three heating devices, as described in a recent paper [62]. For completeness, a short description of these devices is given below. The first primary heating device consists of a number of surface heaters of 17 kW in total that are installed around the outer surfaces of the three perpendicularly-aligned cylindrical pipes and four pressure release pipes (see Fig. 1). These surface heaters slowly heat up the cruciform burner via thermal conduction, eventually balancing heat losses to retain a fixed temperature up to 523 K. Unfortunately, this traditional surface heating method cannot provide a uniform temperature in the domain of experiment, because there is a declining temperature gradient (about 2.5 °C/cm) from the burner heating surface to its center, see Fig. 2 in [62]. In order to solve such a non-uniform temperature problem, the second primary heating device is designed, i.e. each of the two perforate plates is carefully welded by a long narrow serpentine heating strip of a cross sectional area of 2.5 × 2.5 mm ^2^ without blocking any of these 10 mm holes, see purposely transparent part and enlarged perforated plate with heating wires on the right of Fig. 1. This novel design allows us to retain the same turbulence characteristics, while efficiently heating up gas inside the burner due to convection when the counter-rotated fans are turned on. An essentially uniform temperature distribution in the domain of measurements can be created, with the temperature variations being less than ±1 °C, see Fig. 2 in [62]. Such a unique perforated plate heating device is a useful contribution for the study of both gaseous and liquid fuels in high-temperature premixed combustion. The third heating device is a secondary air heater that is used to further speed up the heating procedure.

**Fig. 2:**
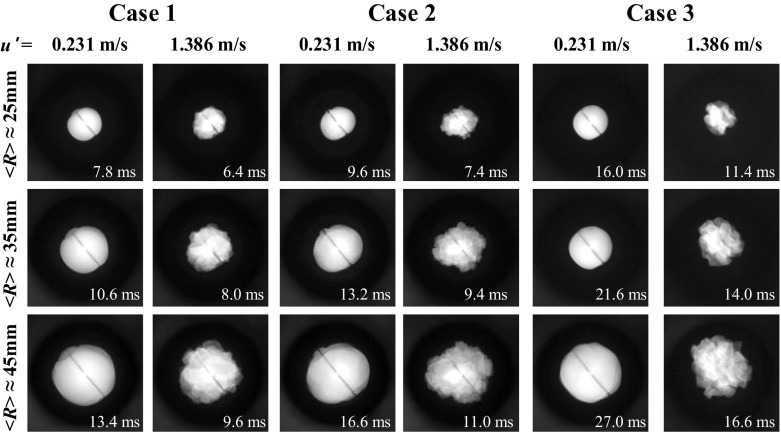
Typical instantaneous direct flame images associated with the mean flame radius $\bar {R}_{f}=25$ mm (the first row), 35 mm (the second row), and 45 mm (the third row), obtained in cases 1 (two left columns), 2 (two middle columns), and 3 (two right columns) at $u^{\prime }=$0.213 m/s (the left sub-column in each case) and 1.386 m/s (the right sub-column). Field of view is 16 × 16 cm ^2^

Both inner and outer chambers are optically accessible for Schlieren and direct flame imaging. As discussed in detail elsewhere [13, 57], both techniques yield essentially the same turbulent flame speeds within experimental uncertainties. In the present work, direct images of centrally-ignited, outwardly-propagating premixed flames were recorded using a high-speed Phantom camera (v711) operated at a frame rate of 11 000 frames/s. Typical instantaneous direct flame images presented in Fig. 2 show that the flames retained the spherical (in the mean) shape and enveloped the same central part of the combustion chamber in all studied cases, thus, indicating, negligible mean and isotropic turbulent flow in the plane of view, as well as negligible buoyancy effects.

To evaluate the mean flame radii $\bar {R}_{f}$ at various instants *t*, the images were processed using a method described in [56, 57], i.e. (i) the images were binarized, (ii) the area $A\left (t \right )$ enveloped by the flame contour was determined for each image, and (iii) the instantaneous flame radius was calculated as follows $\bar {R}_{f}\left (t \right )=\sqrt {A\left (t \right )} \left / \right . \pi $. Subsequently, flame displacement speeds were evaluated using

3 $$ S_{t}\left( t \right)=\frac{1}{\sigma} \frac{d\bar{R}_{f}}{dt}  $$

where, in line with the common practice, a factor of *σ* ^−1^ was invoked in order to allow for unburned gas flow induced due to thermal expansion. It is worth stressing that such a method of measuring $\bar {R}_{f}\left (t \right )$ and $S_{t}\left (t \right )$ by processing Schlieren or direct flame images obtained from expanding statistically spherical premixed turbulent flames is widely accepted and is used not only by the present [13, 56, 57], but also by many other research groups, e.g. see [8, 12, 16, 17] and earlier papers reviewed in [4]. It is also worth stressing that the present work aims at comparing dependencies of $\bar {R}_{f}\left (t \right )$, obtained in cases 2 and 3 using exactly the same experimental technique, with images of flames 2 and 3 looking similar at similar $\bar {R}_{f}\left (t \right )$, see Fig. 2.

Henceforth, symbol *S*_*t*_ designates turbulent flame displacement speed evaluated with respect to unburned gas using Eq. 3. The observed turbulent flame displacement speed *S*_*t*, *b*_, which is evaluated with respect to burned gas, is simply equal to ${d\bar {R}_{f}} \left / \right . {dt}$ or *σ* *S*_*t*_. Even if *S*_*t*_ depends weakly on the density ratio under conditions of the present experiments, as will be shown in the next section, the observed speed *S*_*t*, *b*_ is increased by *σ* due to a more pronounced expansion of lighter products.

Nevertheless, it is *S*_*t*_ evaluated using Eq. 3, rather than $S_{t,b}={d\bar {R}_{f}} \left / \right . {dt}=\sigma S_{t}$, that is commonly used [8, 12, 13, 16, 17] to characterize burning rate in expanding statistically spherical turbulent premixed flames. In particular, Bradley et al. [63] have thoroughly argued that turbulent burning (consumption) velocity is properly characterized by the flame displacement speed defined by Eq. 3 provided that *S*_*t*_ is multiplied with a pre-factor, which does not depend on mixture composition (and, hence, on *σ*). Applicability of the widely accepted Eq. 3 to investigating eventual effects of *σ* on turbulent flame speed is further discussed in the Appendix.

## Results

**Fig. 3:**
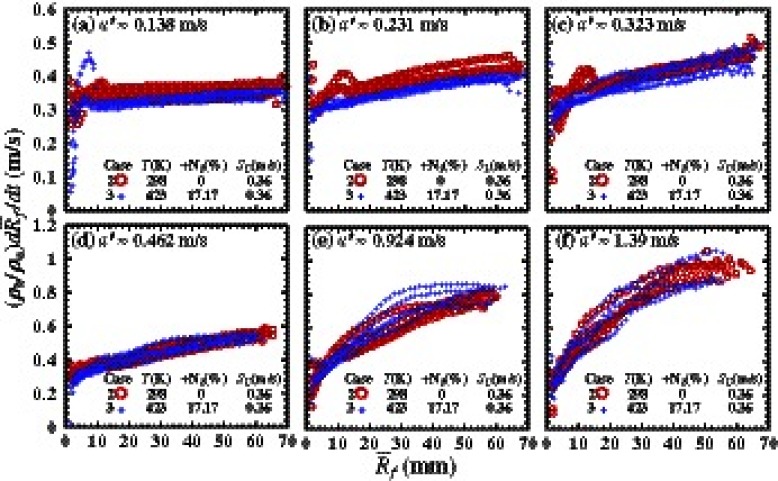
Dependencies of the turbulent flame displacement speed $S_{t}=\sigma ^{-1}{d\bar {R}_{f}} \left / \right . {dt}$ on the mean flame radius $\bar {R}_{f}$, measured at six different $u^{\prime }$. *Circle* and *plus symbols* represent data obtained in cases 2 and 3 characterized by the same laminar flame speed, but different density ratios *σ*. In each case, data obtained in five runs are reported

Figure 3 shows dependencies of the turbulent flame displacement speed $S_{t}=\sigma ^{-1}{d\bar {R}_{f}} \left / \right . {dt}$ on the mean flame radius $\bar {R}_{f}$, measured in cases 2 and 3 at six different $u^{\prime }$. At each $u^{\prime }$, data obtained from five identical runs are reported.

First, after an initial phase associated with the spark discharge influence on the flame kernel, the data obtained from different runs collapse to a mean curve, which indicates an increase in the flame displacement speed with the flame kernel radius. At a higher $u^{\prime }$, both the scatter of the data around mean curves and the increase in *S*_*t*_ with $\bar {R}_{f}$ are more pronounced. Because these trends are beyond the scope of the present study, we refer the interested reader to [4, 64] where the growth of the flame displacement speed was well predicted in RANS simulations or to [65, 66] where two simplified models of the growth of *S*_*t*_ were developed and validated. Another approach to modeling this effect was put forward by Chaudhuri et al. [67]. According to these papers, the growth of *S*_*t*_ results from (i) the inherent development of a premixed turbulent flame [64–67] and (ii) weakening of the reduction effect of the curvature of the mean flame brush on the mean flame displacement speed [64–66]. Because the magnitude of the latter effect scales as $\delta _{t} \left / \right . \bar {R}_{f}$ [65, 66] and the mean flame brush thickness *δ*_*t*_ is increased by $u^{\prime }$ [4], the increase in ${dS_{t}} \left / \right . {d\bar {R}_{f}}$ by $u^{\prime }$, shown in Fig. 3, is consistent with the model developed in [64–66]. Indeed, if $u^{\prime }$ is low (high), with all other things being equal, then, $\delta _{t} \left / \right . \bar {R}_{f}$ is small (large), the reduction effect is weakly (well) pronounced, and *S*_*t*_ is close to (far from) its value associated with the statistically planar premixed flame. Accordingly, the growth of *S*_*t*_ due to the weakening of the reduction effect is weakly (well) pronounced.

Second, Fig. 3 shows that data obtained in cases 2 and 3 are very close to one another in spite of substantially different *σ* in the two cases. To be more specific, in very weak turbulence, i.e. $u^{\prime } \quad =$ 0.138 or 0.231 m/s, the flame displacement speeds obtained in case 2 characterized by a larger *σ* are slightly higher than in case 3. The opposite trend is observed at $u^{\prime } \quad =$ 0.924 m/s in the medium range of $\bar {R}_{f}$, but these data are more scattered. At three other values of $u^{\prime }$, an effect of *σ* on the flame displacement speed is not pronounced.

**Fig. 4:**
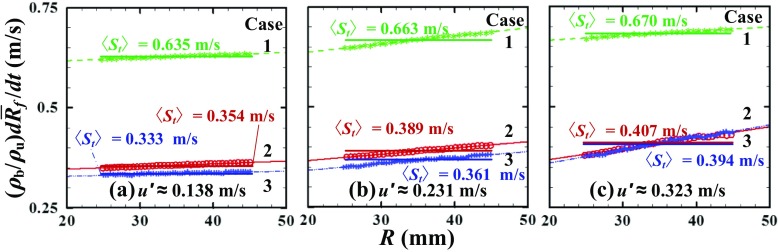
Same data as in Fig. 3 but averaged over those five runs in each case. Case 1 is also included. Thin straight lines fit the measured data shown in symbols. Thick horizontal straight lines show the mean turbulent flame displacement speed 〈*S*_*t*_〉, i.e. the linear slope of $\langle R_{f}\rangle \left (t \right ) $ at 25 mm <〈*R*_*f*_〉<45 mm, divided with *σ*

A weak (if any) effect of the density ratio on the flame displacement speed can also be shown by presenting the same data in another form used e.g. in [56, 57]. To do so, first, five dependencies of $\bar {R}_{f}\left (t \right )$ obtained under statistically the same conditions (i.e. the dependencies reported in Fig. 3) were averaged in order to obtain a dependence of a mean flame radius 〈*R*_*f*_〉 on time. Second, each obtained curve $\langle R_{f}\rangle \left (t \right )$ was differentiated and the results divided with *σ* are plotted in symbols in Fig. 4. Third, each set of symbols was fitted with a straight line within a range of 〈*R*_*f*_〉∈ [25, 45] mm, see thin lines in Fig. 4. Fourth, the mean flame displacement speed 〈*S*_*t*_〉 see thick horizontal straight lines in Fig. 4, was set equal to a value given by the fitting straight line at 〈*R*_*f*_〉 = 35 mm.

**Fig. 5:**
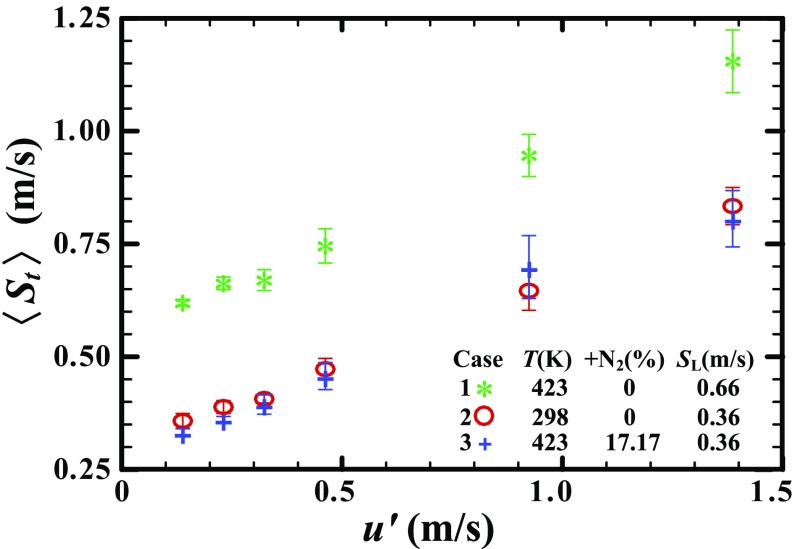
Dependencies of the mean turbulent flame displacement speed 〈*S*_*t*_〉 on $u^{\prime }$

Similar to the raw data on $S_{t}\left (\bar {R}_{f} \right )$, plotted in Fig. 3, which is the major result of the present work, dependencies of 〈*S*_*t*_〉 on $u^{\prime }$, reported in Fig. 5 do not show a significant effect of the density ratio on 〈*S*_*t*_〉. A small difference in 〈*S*_*t*, 2_〉 and 〈*S*_*t*, 2_〉 observed in weak turbulence is within the range of experimental uncertainties (i.e. the rms scatter of the data from different runs) indicated with vertical bars. In case 1, $\langle S_{t,2} \rangle \left (u^{\prime } \right )$ is significantly higher than in cases 2 and 3, because *S*_*L*, 1_ is larger than *S*_*L*, 2_ or *S*_*L*, 3_

Figure 5 also indicates that the lowest 〈*S*_*t*_〉 obtained at $u^{\prime }=$ 0.138 m/s is close to *S*_*L*_ in all three cases studied. This result is consistent with very weak wrinkling of flame kernel in the images obtained at $u^{\prime }=$ 0.231 m/s and shown in Fig. 2.

**Fig. 6:**
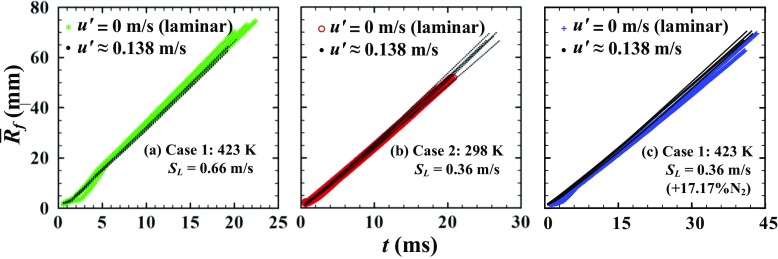
Comparison of $\bar {R}_{f}(t)$ obtained from laminar (three runs) and weakly turbulent (five runs) flames

In order to more carefully check whether or not *S*_*t*_ is sensitive to weak turbulence, raw data on $\bar {R}_{f}\left (t \right )$ obtained from laminar and weakly turbulent flames, with all other things being equal, are compared in Fig. 6. In each case 1, 2, or 3 data obtained in three (five) runs are reported for laminar (turbulent) flames. The same three and five curves were earlier used to evaluate *S*_*L*_ and 〈*S*_*t*_〉 at $u^{\prime }=$ 0.138 m/s, respectively, and the same five curves are plotted in another form in cases 2 and 3 in Fig. 3a. Figure 6 does not indicate substantial difference between data obtained from the laminar and turbulent flames. Due to the lack of a notable influence of weak turbulence on the speeds of the studied flames, a simple linear interpolation of the data on 〈*S*_*t*_〉, shown in Fig. 5, to $u^{\prime }\to 0$ yields values lower than *S*_*L*_

**Fig. 7:**
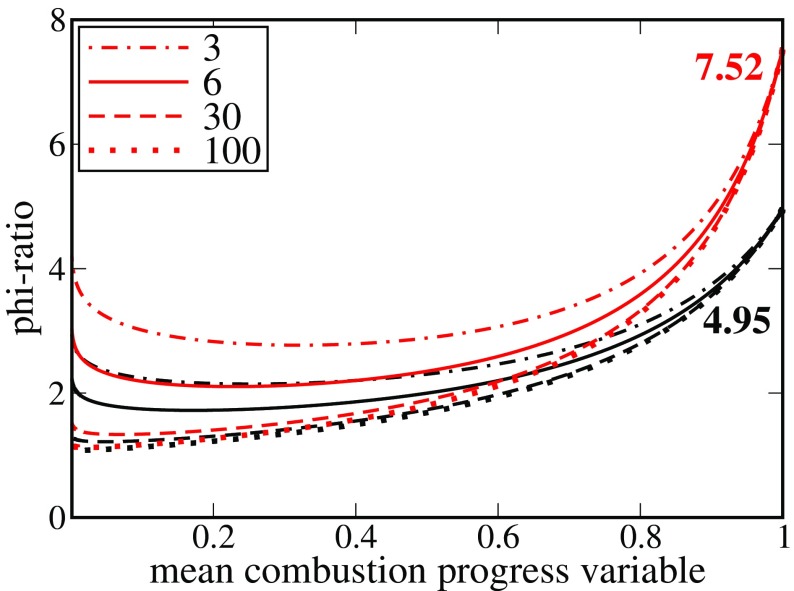
Dependencies of the *φ*-factor on the reference value of the mean combustion progress variable, computed using Eqs. A.6–A.9 at *σ* = 7.52 (*red lines*) and *σ* = 4.95 (*black lines*) and various ratios of $r_{f} \left / \right . \delta _{t}$, specified in legends

It is worth remembering that approximately equal turbulent and laminar flame speeds were earlier obtained from weakly turbulent V-shaped flames [??68, Fig. 7] and from weakly turbulent expanding statistically spherical flames at $\bar {R}_{f}$ as large as 60 mm [69, Fig. 6].

## Discussion

At first glance, the primary experimental result of the present work, i.e. evidence that the flame displacement speed *S*_*t*_ defined by Eq. 3 is not notably increased by the density ratio even if the turbulence is weak ($u^{\prime }<S_{L})$, appears to challenge concepts that assume that combustion-induced thermal expansion substantially increases turbulent flame speed due to (i) flame-generated turbulence, (ii) the local DL instability, or (iii) an increase in scalar dissipation rate due to dilatation. Indeed, the present authors do not see how concept (i) or (iii) could be consistent with the present experimental data. However, Figs. 3, 4, 5 and 6 neither challenge concept (ii) nor contradict to DNS studies [45–47, 70] that indicated substantial influence of the DL mechanism on premixed turbulent combustion.

Indeed, first, a recent 2D DNS investigation [47] of weakly turbulent premixed flames subject to the DL instability also shows that the density ratio weakly affects turbulent flame speed, e.g. an increase in *σ* by a factor of three (from two to six) results in increasing $S_{t} \left / \right . S_{L}$ by 10 % only, see Fig. 11 in the cited paper.

Second, there is an effect that can mitigate the local DL instability for the flame configuration investigated in the present experiments. The point is that, due to stabilizing influence of molecular transport and flame stretching, expanding spherical laminar flames characterized by *L* *e* > *L* *e*_*cr*_, where *L* *e*_*cr*_ < 1, are well known to be stable if the flame kernel radius is less than a sufficiently large critical value *R*_*cr*_ [71–73]. For instance, recent experiments with the stoichiometric laminar propane-air flames [74] yielded *R*_*cr*_ as large as 200 mm. The laminar stoichiometric CH_4_/O_2_/N_2_ flames studied by us were also stable due to the stretch effect. Accordingly, one could assume that the DL instability does not accelerate turbulent burning due to stabilizing effect of the mean curvature and stretching of the mean flame brush under conditions of the present experiments.

Moreover, in a turbulent flow, the stabilizing stretch-effect can manifest itself not only in global mitigation of the DL instability due to the mean curvature of a mean flame surface, but also in local mitigation of the DL instability of flamelets by the local turbulent stretching [50], with even very low stretch rates $\dot {s}$ being efficient. For instance, the aforementioned experimental data [74] show that the stoichiometric propane-air spherical laminar flame is stabilized by the normalized curvature $2\kappa _{u} \left / \right . \left (R_{f}S_{L} \right )$ and stretch rate $\dot {s}\kappa _{u} \left / \right . {S_{L}^{2}}$ as low as 0.00055 and 0.0044, respectively. Therefore, even in a weakly turbulent flow, positively curved and stretched flamelets can be locally stable with respect to the DL instability.

Thus, as far as a role played by the DL instability in premixed turbulent combustion is concerned, the experimental data presented in Figs. 3 and 6 should not be interpreted to show that the instability weakly affects turbulent flame speed in a general case. Nevertheless, even if independence of *S*_*t*_ on *σ* is documented in a particular case of expanding statistically spherical flames, this result has a value. The value is associated with the facts that (i) an expanding statistically spherical premixed turbulent flame is the best laboratory model for investigating combustion in Spark Ignition (SI) engines and, (ii) due to mixture compression by a piston, the engine flame is characterized by significantly lower density ratio when compared to a typical hydrocarbon-air flame under the room conditions. Accordingly, the present results imply a minor effect of the density ratio (and, in particular, the DL instability, see Fig. 6, which does not indicate an influence of weak turbulence on the speed of flame kernel growth when compared to the counterpart laminar case) on flame displacement speed in SI engines and support simulations of the engine combustion using models that (i) do not allow for effects of the density ratio and DL instability on *S*_*t*_ and (ii) have been validated against experimental data obtained under the room conditions, i.e. at higher *σ*.

## Conclusions

A method is suggested in order to experimentally investigate an influence of the density ratio *σ* on turbulent flame speed by retaining the laminar flame speed *S*_*L*_ unchanged. The method consists of (i) preheating a flammable mixture in order to increase *S*_*L*_, but to decrease *σ*, and (ii) diluting the preheated mixture in order to further decrease *σ*, but to reduce *S*_*L*_ back to the initial value.

Experiments at 298 K and 423 K were conducted in a modified dual-chamber explosion facility that applied various heating devices including a pair of perforated plate heaters. This novel design of perforated plate heaters allowed us to retain the same turbulence characteristics, while efficiently heating up gas inside the burner due to convection when the counter-rotated fans were turned on. Thus, an essentially uniform temperature distribution was created in the domain of measurements.

While the density ratio was varied in a sufficiently narrow range (34 %) under conditions of the present experiments, it is worth remembering that, to the best of the present authors’ knowledge, turbulent flame speed has not yet been experimentally obtained from mixtures characterized by the same laminar flame speed, but substantially different density ratios.

Experimental data obtained from expanding statistically spherical stoichiometric CH_4_/O_2_/N_2_ flames at various $u^{\prime }$ and *T*_*u*_ = 298 or 423 K do not indicate a significant effect of *σ* on the flame displacement speed *S*_*t*_ defined by Eq. 3.

Moreover, it is found that *S*_*t*_ measured at ${u^{\prime }} \left / \right . S_{L}\le $ 0.4 is close to *S*_*L*_ in all three investigated cases.

## Figures and Tables

**Fig. 8:**
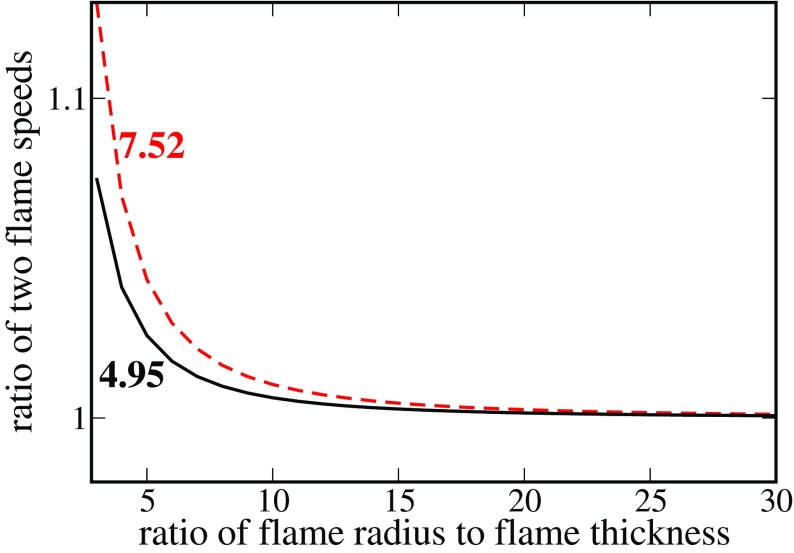
Dependencies of a ratio of the theoretical flame speed $S_{t}={dr_{f}} \left / \right . {dt}-U_{u}$ [82, 83] to the flame displacement speed $S_{t}=\sigma ^{-1}{dr_{f}} {\left / \right . } {dt}$ on the normalized flame radius $r_{f} \left / \right . \delta _{t}$, computed using Eqs. A.6–A.9 at *σ* = 7.52 (*red dashed line*) and *σ* = 4.95 (*black solid line*)

## Appendix

Let us consider a premixed turbulent flame with a self-similar mean structure, i.e.

A.1 $$ \bar{\rho} \left( x,t \right)=\bar{\rho} \left( \xi \right),\qquad\quad\xi =\frac{x-x_{f}\left( t \right)}{\delta_{t}\left( t \right)}  $$

where $\bar {\rho } $ is the Reynolds-averaged density, $x_{f}\left (t \right )$ is the coordinate of a mean flame surface, $\delta _{t}\left (t \right )$ is the mean flame brush thickness, and the *x*-axis is normal to the mean flame surface. As reviewed elsewhere [4, 75–77], such an assumption holds very well for various premixed turbulent flames, including the spherical ones, e.g. [78, 79].

In the statistically spherical case, the Favre-averaged continuity equation reads

A.2 $$ \frac{\partial \bar{\rho} }{\partial t}+\frac{1}{r^{2}}\frac{\partial} {\partial r}\left( \bar{\rho} r^{2}\tilde{v} \right)=0  $$

where $\tilde {v}\left (r,t \right )=\overline {\rho v} \left / \right . \bar {\rho } $ is the Favre-averaged flow velocity in the radial direction. Substitution of Eq. A.1 with *x* = *r* into Eq. A.2 yields

A.3 $$ -\left( \frac{S_{b}}{\delta_{t}}+\frac{\xi} {\delta_{t}}\frac{d\delta_{t}}{dt} \right)\frac{d\bar{\rho} }{d\xi} +\frac{1}{r^{2}}\frac{\partial} {\partial r}\left( \bar{\rho} r^{2}\tilde{v} \right)=0,  $$

where $S_{b}={dr_{f}} \left / \right . {dt}$. Integration of Eq. A.3 from 0 to *r*, followed by integration by parts results in

A.4 $$\begin{array}{@{}rcl@{}} \bar{\rho} r^{2}\tilde{v}&=&{\int\limits_{0}^{r}} {y^{2}\left( S_{b}+\frac{y-r_{f}}{\delta_{t}}\frac{d\delta_{t}}{dt} \right)\frac{d\bar{\rho} }{dy}dy} \\&&\qquad=\bar{\rho} r^{2}\left( S_{b}+\frac{r-r_{f}}{\delta_{t}}\frac{d\delta_{t}}{dt} \right)-{\int\limits_{0}^{r}} {\bar{\rho} \left[ 2y\left( S_{b}+\frac{y-r_{f}}{\delta_{t}}\frac{d\delta_{t}}{dt} \right)+\frac{y^{2}}{\delta_{t}}\frac{d\delta_{t}}{dt} \right]dy}\\ \end{array} $$

Consequently, the speed $S_{t}^{\ast } \left [ r=r_{f}\left (t \right ),t \right ]$ of a mean flame surface $\bar {\rho } \left [ r=r_{f}\left (t \right ),t \right ]=\rho ^{\ast } $ with respect to the local radial gas velocity $\tilde {v}=\tilde {v}\left [ r=r_{f}\left (t \right ),t \right ]$ is equal to

A.5 $$ S_{t}^{\ast} =S_{b}-\tilde{v}=\frac{2S_{b}}{\bar{\rho} r^{2}}{\int\limits_{0}^{r}} {\bar{\rho} ydy} -\frac{r-r_{f}}{\delta_{t}}\frac{d\delta_{t}}{dt}+\frac{1}{\bar{\rho} r^{2}}\frac{d\delta_{t}}{dt}{\int\limits_{0}^{r}} {\bar{\rho} \frac{3y^{2}-2yr_{f}}{\delta_{t}}dy.}  $$

If the flame brush thickness is constant, then,

A.6 $$ S_{t}^{\ast} =S_{b}-\tilde{v}=\frac{2S_{b}}{\bar{\rho} {r_{f}^{2}}}{\int\limits_{0}^{r}}_{f} {\bar{\rho} ydy} =\frac{\rho_{u}}{\bar{\rho} \left( r_{f} \right)} \frac{\langle \rho\rangle} {\rho_{u}}\frac{dr_{f}}{dt}=\frac{\rho_{u}}{\bar{\rho} \left( r_{f} \right)} \frac{\langle \rho\rangle} {\rho_{b}}\frac{1}{\sigma} \frac{dr_{f}}{dt}=\frac{\varphi} {\sigma} \frac{dr_{f}}{dt}=\varphi S_{t}  $$

where $S_{t}=\sigma ^{-1}{dr_{f}} \left / \right . {dt}$,

A.7 $$ \langle \rho\rangle=\frac{2}{{r_{f}^{2}}}{\int\limits_{0}^{r}}_{f} {\bar{\rho} ydy}  $$

and $\varphi =\left (\rho _{u} \left / \right . \bar {\rho } \right )\left (\langle \rho \rangle {\left / \right . } \rho _{b} \right )>1$, because both $\rho _{u} \left / \right . {\bar {\rho } \left (r_{f} \right )}>1$ and $\langle \rho \rangle {\left / \right .} \rho _{b}>1$ for a finite flame-brush thickness. In the case of an infinitely thin flame brush, $\bar {\rho } \left (r_{f} \right )=\rho _{u}$, 〈*ρ*〉 = *ρ*_*b*_, and *φ* = 1.

Thus, the use of the mean density 〈*ρ*〉 makes $S_{t}^{\ast } $ larger than *S*_*t*_. To evaluate this difference and its dependence on the density ratio, the mean flame brush thickness $\delta _{t}\left (t \right )$ and the mean density profile $\bar {\rho } \left (r \right )$ should be known, but they were not measured in the present experiments. Nevertheless, estimates can be performed invoking results of many other experiments that, in particular, showed that variations of the mean combustion progress variable $\bar {c}$ along the normal to the mean flame brush are well parameterized with the complementary error function [4, 76–78]

A.8 $$ \bar{c}=\frac{1}{2}\text{erfc}\left( \xi \sqrt \pi \right)=\frac{1}{\sqrt \pi} \int\limits_{\xi \sqrt \pi}^{\infty} {\exp\left( -\mu^{2} \right)d\mu}  $$

Substitution of this parameterization and the well-known BML equation [80]

A.9 $$ \bar{\rho} =\rho_{u}\left( 1-\bar{c} \right)+\rho_{b}\bar{c}  $$

into Eqs. A.6 and A.7 allows us to model dependence of the *φ*-factor on *σ*, $r_{f} \left / \right . \delta _{t}$, and a reference value *c* ^∗^of the mean combustion progress variable associated with the mean flame surface, i.e. $\bar {c}\left [ r=r_{f}\left (t \right ),t \right ]=c^{\ast } $.

Figure 7 indicates that the *φ*-factor (i) is substantially larger than unity if a ratio of $r_{f} \left / \right . \delta _{t}$ is moderate (see dotted-dashed and solid lines), (ii) is reduced and tend to unity on the unburned flame side when $r_{f} \left / \right . \delta _{t}$ is increased (see dashed and dotted lines), and (iii) is increased by the density ratio (cf. red and black lines).

Images reported in the case of $u^{\prime }=0.231$ m/s in Fig. 2 show that the flames expand from $\bar {R}_{f}=25$ mm to $\bar {R}_{f}=45$ mm during intervals of 7.8-13.4 ms in case 2 and 16.0-27.0 ms in case 3. Variations in the mean flame brush thickness *δ*_*t*_ during these time intervals can be estimated invoking the following simple relation $\delta _{t}\left (t \right )\approx \sqrt {2\pi } u't$, which is well supported by various experimental data [4, 76, 77], including data obtained from expanding spherical flames [78, 79]. Accordingly, during these time intervals, a ratio of $\bar {R}_{f} \left / \right . \delta _{t}$ is about six and three in cases 2 and 3, respectively. Results calculated in such two cases are plotted in red solid and black dotted-dashed lines, respectively, in Fig. 7. In each of the two cases, the influence of *σ* on *φ* is substantial. However, it is worth remembering that, due to a stronger thermal expansion, flame kernel 2 grows faster and, hence, is characterized by a larger $\bar {R}_{f} \left / \right . \delta _{t}$ when compared to flame kernel 3, with an increase in $\bar {R}_{f} \left / \right . \delta _{t}$ reducing *φ*. Under conditions of the present study, the two effects counterbalance one another almost completely in the range of $\bar {c}<0.5$ (cf. curves shown in red solid and black dotted-dashed lines). Because $\bar {R}_{f}$ obtained from direct flame images is associated with a low $\bar {c}$, the discussed model results imply that the values of the *φ*-factor are almost the same in cases 2 and 3 under conditions of the present study, thus, justifying the use of Eq. 3 in order to assess whether or not the density ratio affects the relative flame speed $S_{t}^{\ast }\left (\bar {c}\le 0.5 \right )$.

Moreover, the above estimates address flames with a constant *δ*_*t*_, whereas expanding spherical flames are characterized by the growing mean flame brush thickness [4, 63, 78, 79], with the growth of *δ*_*t*_ reducing the difference in $S_{t}^{\ast } $ and the flame speed $S_{t}=\sigma ^{-1}{dr_{f}} \left / \right . {dt}$. Indeed, Eqs. A.5 and A.6 read

A.10 $$ S_{t}^{\ast} =\varphi S_{t}-\frac{\psi} {\bar{\rho} r^{2}}\frac{1}{\delta_{t}}\frac{d\delta_{t}}{dt} $$

where

A.11 $$ \psi =\bar{\rho} \left( r^{3}-r^{2}r_{f} \right)-{\int\limits_{0}^{r}} {\bar{\rho} \left( 3y^{2}-2yr_{f} \right)dy.} $$

In the case of a finite flame brush thickness and *r*≥*r*_*f*_, the function *ψ* is positive, because (i) *ψ* = 0 if $\bar {\rho } \left (r<r_{f} \right )=\bar {\rho } \left (r=r_{f} \right )$, but $\bar {\rho } \left (r<r_{f} \right )<\bar {\rho } \left (r=r_{f} \right )$ for an expanding statistically spherical flame of a finite thickness.

Furthermore, a definition of turbulent flame speed is still an issue and variously determined flames speeds can be found in the literature, as reviewed elsewhere [4, 76, 77, 81]. In particular, there is no evidence that $S_{t}^{\ast } $ yielded by Eq. A.10 characterizes turbulent burning rate better than the flame speed determined using Eq. 3. On the contrary, as theoretically argued elsewhere [82, 83], in order for a flame speed *S*_*t*_ to properly characterize turbulent burning rate, i.e. to be equal to turbulent consumption velocity *U*_*t*_, this *S*_*t*_ should be equal to ${dr_{f}} \left / \right . {dt}-U_{u}$, rather than ${dr_{f}} \left / \right . {dt}-\tilde {v}\left [ r=r_{f}\left (t \right ),t \right ]$, with *U*_*u*_ being evaluated by extrapolating the mean velocity distribution in the unburned gas to a surface of $\bar {c}\left (r,t \right )=0.5$. Because *U*_*u*_ is larger than $\tilde {v}=\tilde {v}\left [ r=r_{f}\left (t \right ),t \right ]$, which peaks at a low $\bar {c}<0.5$, the theoretical *S*_*t*_ should be smaller than $S_{t}^{\ast } $.

Indeed, using Eqs. A.6–A.9, the unburned gas velocity $\tilde {v}_{u}=\tilde {v}\left (\bar {c}\to 0 \right )$ can easily be computed in the case of a constant *δ*_*t*_, followed by evaluation of (i) the extrapolated velocity $U_{u}=\tilde {v}_{u}\left [ r_{u} \left / \right . {r_{f}\left (\bar {c}=0.5 \right )} \right ]^{2}$ and (ii) a ratio of the theoretical flame speed $S_{t}={dr_{f}} \left / \right . {dt}-U_{u}$ (or the turbulent consumption velocity *U*_*t*_) to the flame displacement speed $S_{t}=\sigma ^{-1}{dr_{f}} \left / \right . {dt}$. Here, *r*_*u*_ is the radius of a mean surface associated with $\tilde {v}_{u}$. Results plotted in Fig. 8 indicate that this flame-speed ratio is close to unity and is weakly affected by the density ratio, thus, supporting the use of Eq. 3 for the goals of the present work. It is worth noting that these estimates that $U_{t} \left / \right . S_{t}$ (i) is larger than unity, but, nevertheless, (ii) is sufficiently close to unity, and (iii) depends weakly on the density ratio agree very well with results of analysis of experimental data by Bradley et al. [63]. Therefore, the flame speed defined by Eq. 3 appears to be an appropriate quantity for investigating eventual influence of the density ratio on turbulent burning rate.

## Footnotes

1 Due to stabilizing effect of molecular transport, a laminar premixed flame is stable with respect to infinitesimal perturbations with wavelengths shorter than *λ*_*n*_, which is controlled by the mixture composition, temperature, and pressure [39].

2 It is worth remembering that a stretched laminar flame is characterized by an infinite set of differently defined Markstein lengths [39, 53] and selection of *ℒ* that is most appropriate for modeling turbulent combustion is still an open issue.

## References

[1] Andrews GE, Bradley D (1972). Determination of burning velocities: a critical review. Combust. Flame.

[2] Abdel-Gayed RG, Bradley D, Lawes M (1987). Turbulent burning velocities: a general correlation in terms of straining rates. Proc. R. Soc. London A.

[3] Gülder ÖL (1990). Turbulent premixed flame propagation models for different combustion regimes. Symp. (Int.) Combust..

[4] Lipatnikov AN, Chomiak J (2002). Turbulent flame speed and thickness: phenomenology, evaluation, and application in multi-dimensional simulations. Prog. Energy Combust. Sci..

[5] Kobayashi H, Seyama K, Hagiwara H, Ogami Y (2005). Burning velocity correlation of methane/air turbulent premixed flames at high pressure and high temperature. Proc. Combust. Inst..

[6] Filatyev SA, Driscoll JF, Carter CD, Donbar JM (2005). Measured properties of turbulent premixed flames for model assessment, including burning velocities, stretch rates, and surface densities. Combust. Flame.

[7] Lawn CG, Schefer RW (2006). Scaling of premixed turbulent flames in the corrugated regime. Combust. Flame.

[8] Bradley D, Lawes M, Mansour MS (2011). Correlation of turbulent burning velocities of ethanol-air, measured in a fan-stirred bomb up to 1.2 MPa. Combust. Flame.

[9] Daniele S, Jansohn P, Mantzaras J, Boulouchos K (2011). Turbulent flame speed for syngas at gas turbine relevant conditions. Proc. Combust. Inst..

[10] Tamadonfar P, Gülder ÖL (2014). Flame brush characteristics and burning velocities of premixed turbulent methane/air Bunsen flames. Combust. Flame.

[11] Troiani G, Creta F, Matalon M (2015). Experimental investigation of Darrieus-Landau instability effects on turbulent premixed flames. Proc. Combust. Inst..

[12] Wu F, Saha A, Chaudhuri S, Law CK (2015). Propagation speeds of expanding turbulent flames of C4 to C8 n-alkanes at elevated pressures: experimental determination, fuel similarity, and stretch-affected local extinction. Proc. Combust. Inst..

[13] Shy, S.S., Liu, C.C., Lin, J.Y., Chen, L.L., Lipatnikov, A.N., Yang, S.I.: Correlations of high-pressure lean methane and syngas turbulent burning velocities: effects of turbulent Reynolds, Damköhler, and Karlovitz numbers. Proc. Combust. Inst. **35**, 1509–1516 (2015)

[14] Venkateswaran P, Marshall A, Seitzman J, Lieuwen T (2015). Scaling turbulent flame speeds of negative Markstein length fuel blends using leading points concepts. Combust. Flame.

[15] Kheirkhah S, Gülder ÖL (2015). Consumption speed and burning velocity in counter-gradient and gradient diffusion regimes of turbulent premixed combustion. Combust. Flame.

[16] Burluka AA, Gaughan RG, Griffiths JF, Mandilas C, Sheppard CGW, Woolley R (2016). Turbulent burning rates of gasoline components, Part 1 - Effect of fuel structure of C6 hydrocarbons. Fuel.

[17] Goulier, J., Comandini, A., Halter, F., Chaumeix N.: Experimental study on turbulent expanding flames of lean hydrogen/air mixtures. Proc. Combust. Inst **36**. in press, available online http://www.sciencedirect.com/science/article/pii/S1540748916301328

[18] Wabel, T.M., Skiba, A.W., Driscoll, J.F.: Turbulent burning velocity measurements: Extended to extreme levels of turbulence. Proc. Combust. Inst **36**. in press, available online http://www.sciencedirect.com/science/article/pii/S1540748916304023

[19] Günther R (1983). Turbulence properties of flames and their measurement. Prog. Energy Combust. Sci..

[20] Lipatnikov AN, Chomiak J (2010). Effects of premixed flames on turbulence and turbulent scalar transport. Prog. Energy Combust. Sci..

[21] Sabelnikov VA, Lipatnikov AN (2017). Recent advances in understanding of thermal expansion effects in premixed turbulent flames. Annu. Rev. Fluid Mech..

[22] Karlovitz B, Denniston DW, Wells FE (1951). Investigation of turbulent flames. J. Chem. Phys..

[23] Scurlock AC, Grover JH (1953). Propagation of turbulent flames. Symp. (Int.) Combust..

[24] Poludnenko AY (2015). Pulsating instability and self-acceleration of fast turbulent flames. Phys. Fluids.

[25] Veynante D, Trouvé A, Bray KNC, Mantel T (1997). Gradient and counter-gradient scalar transport in turbulent premixed flames. J. Fluid Mech..

[26] Mura A, Champion M (2009). Relevance of the Bray number in the small-scale modeling of turbulent premixed flames. Combust. Flame.

[27] Lipatnikov AN (2011). Transient behavior of turbulent scalar transport in premixed flames. Flow Turbul. Combust..

[28] Zimont VL, Biagioli F (2002). Gradient, counter-gradient transport and their transition in turbulent premixed flames. Combust. Theory Modelling.

[29] Lecocq G, Richard S, Colin O, Vervisch L (2010). Gradient and counter-gradient modeling in premixed flames: theoretical study and application to the LES of a lean premixed turbulent swirl burner. Combust. Sci. Technol..

[30] Sabelnikov VA, Lipatnikov AN (2011). A simple model for evaluating conditioned velocities in premixed turbulent flames. Combust. Sci. Technol..

[31] Robin V, Mura A, Champion M (2012). Algebraic models for turbulent transports in premixed flames. Combust. Sci. Technol..

[32] Lipatnikov AN, Sabelnikov VA, Nishiki S, Hasegawa T, Chakraborty N (2015). DNS Assessment of a simple model for evaluating velocity conditioned to unburned gas in premixed turbulent flame. Flow Turbul. Combust..

[33] Sabelnikov VA, Lipatnikov AN (2013). Transition from pulled to pushed premixed turbulent flames due to countergradient transport. Combust. Theory Modelling.

[34] Sabelnikov VA, Lipatnikov AN (2015). Transition from pulled to pushed fronts in premixed turbulent combustion: theoretical and numerical study. Combust. Flame.

[35] Landau LD, Lifshitz EM (1987). Fluid mechanics.

[36] Kuznetsov VR, Sabelnikov VA (1990). Turbulence and combustion.

[37] Paul RN, Bray KNC (1996). Study of premixed turbulent combustion including Landau-Darrieus instability effects. Symp. (Int.) Combust..

[38] Chaudhuri S, Akkerman V, Law CK (2011). Spectral formulation of turbulent flame speed with consideration of hydrodynamic instability. Phys. Rev. E.

[39] Clavin P (1985). Dynamical behavior of premixed flame fronts in laminar and turbulent flows. Prog. Energy Combust. Sci..

[40] Bychkov, V.: Importance of the Darrieus-Landau instability for strongly corrugated turbulent flames. Phys. Rev. E **68**, 066304 (2003) 10.1103/PhysRevE.68.066304 14754312

[41] Kolla H, Rogerson JW, Chakraborty N, Swaminathan N (2009). Scalar dissipation rate modeling and its validation. Combust. Sci. Technol..

[42] Chakraborty, N., Champion, M., Mura, A., Swaminathan, N.: Scalar-dissipation-rate approach. In: Swaminathan, N, Bray, K. N. C. (eds.) Turbulent Premixed Flames, pp 76–102. Cambridge University Press, Cambridge (2011)

[43] Burluka AA, Griffiths JF, Liu K, Orms M (2009). Experimental studies of the role of chemical kinetics in turbulent flames. Combust. Explos. Shock Waves.

[44] Treurniet TC, Nieuwstadt FTM, Boersma BJ (2006). Direct numerical simulation of homogeneous turbulence in combination with premixed combustion at low Mach number modelled by the G-equation. J. Fluid Mech..

[45] Lipatnikov AN, Chomiak J, Sabelnikov VA, Nishiki S, Hasegawa T (2015). Unburned mixture fingers in premixed turbulent flames. Proc. Combust. Inst..

[46] Fogla N, Creta E, Matalon M (2015). Effect of folds and pockets on the topology and propagation of premixed turbulent flames. Combust. Flame.

[47] Fogla N, Creta E, Matalon M (2017). The turbulent flame speed for low-to-moderate turbulence intensities: hydrodynamic theory vs. experiments. Combust. Flame.

[48] Smith, G.P., Golden, D.M., Frenklach, M., Moriarty, N.W., Eiteneer, B., Goldenberg, M., Bowman, C.T., Hanson, R.K., Song, S., Gardiner, J.W.C., Lissianski, V.V., Qin, Z: GRI-Mech 3.0 (1999). http://www.me.berkeley.edu/grimech/

[49] Kee, R.J., Crcar, J.F., Smooke, M.D., Miller, J.A.: PREMIX: A Fortran program for modeling steady laminar one-dimensional premixed flames, Sandia report SAND85-8249 Sandia National Laboratories (1985)

[50] Lipatnikov AN, Chomiak J (2005). Molecular transport effects on turbulent flame propagation and structure. Prog. Energy Combust. Sci..

[51] Kee, R.J., Rupley, F.M., Miller, J.A.: CHEMKIN-II: A Fortran chemical kinetics package for the analysis of gas-phase chemical kinetics, Sandia report SAND89-8009 Sandia National Lab (1989)

[52] Lipatnikov AN, Shy SS, Li WY (2015). Experimental assessment of various methods of determination of laminar flame speed in experiments with expanding spherical flames with positive Markstein lengths. Combust. Flame.

[53] Giannakopoulos GK, Gatzoulis A, Frouzakis CE, Matalon M, Tomboulides AG (2015). Consistent definitions of “Flame Displacement Speed” and “Markstein Length” for premixed flame propagation. Combust. Flame.

[54] Bychkov V (1998). Nonlinear equation for a curved stationary flame and the flame velocity. Phys. Fluids.

[55] Bradley D, Lau AKC, Lawes M (1992). Flame stretch rate as a determinant of turbulent burning velocity. Phil. Trans. R. Soc. London A.

[56] Liu CC, Shy SS, Chen HC, Peng MW (2011). On interaction of centrally-ignited, outwardly-propagating premixed flames with fully-developed isotropic turbulence at elevated pressure. Proc. Combust. Inst..

[57] Liu CC, Shy SS, Peng MW, Chiu CW, Dong YC (2012). High-pressure burning velocities measurements for centrally-ignited premixed methane/air flames interacting with intense near-isotropic turbulence at constant Reynolds numbers. Combust. Flame.

[58] Shy SS, Lin WJ, Wei JC (2000). An experimental correlation of turbulent burning velocities for premixed turbulent methane-air combustion. Proc. R. Soc. London, A.

[59] Shy, S.S., I, W.K., Lin, M.L.: A new cruciform burner and its turbulence measurements for premixed turbulent combustion study. Exp. Therm. Fluid Sci. **20**, 105–114 (2000)

[60] Shy SS, Lin WJ, Peng KZ (2000). Highly intensity turbulent premixed combustion: general correlations of turbulent burning velocities in a new cruciform burner. Proc. Combust. Inst..

[61] Yang TS, Shy SS (2005). Two-way interaction between solid particles and homogeneous air turbulence: particle settling rate and turbulence modification measurements. J. Fluid Mech..

[62] Jiang LJ, Shy SS, Li WY, Huang HM, Nguyen MT (2016). High-temperature, high-pressure burning velocities of expanding turbulent premixed flames and their comparison with Bunsen-type flames. Combust. Flame.

[63] Bradley D, Haq MZ, Hicks RA, Kitagawa T, Lawes M, Sheppard CGW, Woolley R (2003). Turbulent burning velocity, burned gas distribution, and associated flame surface definition. Combust. Flame.

[64] Lipatnikov AN, Chomiak J (2000). Transient and geometrical effects in expanding turbulent flames. Combust. Sci. Technol..

[65] Lipatnikov AN, Chomiak J (2004). Application of the Markstein number concept to curved turbulent flames. Combust. Sci. Technol..

[66] Lipatnikov AN, Chomiak J (2007). Global stretch effects in premixed turbulent combustion. Proc. Combust. Inst..

[67] Chaudhuri S, Wu F, Law CK (2013). Scaling of turbulent flame speed for expanding flames with Markstein diffusion considerations. Phys. Rev. E.

[68] Ghenaï, C., Gouldin, F.G., Gökalp, I.: Mass flux measurements for burning rate determination of premixed turbulent flames. Proc. Combust. Inst. **27**, 979–987 (1998)

[69] Bauwens, C.R., Bergthorson, J.M., Dorofeev, S.B.: On the interaction of the Darrieus-Landau instability with weak initial turbulence, Proc. Combust. Inst, **36**. in press, available online http://www.sciencedirect.com/science/article/pii/S1540748916302863

[70] Boughanem H, Trouvé A (1998). The domain of influence of flame instabilities in turbulent premixed combustion. Proc. Combust. Inst..

[71] Bechtold JK, Matalon M (1987). Hydrodynamic and diffusion effects on the stability of spherically expanding flames. Combust. Flame.

[72] Addabbo R, Bechtold JK, Matalon M (2002). Wrinkling of spherically expanding flames. Proc. Combust. Inst..

[73] Bradley, D.: Instabilities and flame speeds in large-scale premixed gaseous explosions. Phil. Trans. R. Soc. London A **357**, 3567–3581 (1999)

[74] Bauwens CR, Bergthorson JM, Dorofeev SB (2015). Experimental study of spherical-flame acceleration mechanisms in large-scale propane-air flames. Proc. Combust. Inst..

[75] Lipatnikov AN, Chomiak J (2000). Dependence of heat release on the progress variable in premixed turbulent combustion. Proc. Combust. Inst..

[76] Driscoll JF (2008). Turbulent premixed combustion: flamelet structure and its effect on turbulent burning velocities. Prog. Energy Combust. Sci..

[77] Lipatnikov AN (2012). Fundamentals of premixed turbulent combustion.

[78] Lipatnikov, A.N., Chomiak, J.: Comment on “Turbulent burning velocity, burned gas distribution, and associated flame surface definition” D. Bradley, M.Z. Haq, R.A. Hicks, T. Kitagawa, M. Lawes, C.G.W. Sheppard, R. Woolley. Combust. Flame **133**, 415 (2003). Combust. Flame 137 (2004) 261–263

[79] Renou B, Mura A, Samson E, Boukhalfa A (2002). Characterization of the local flame structure and flame surface density for freely-propagating premixed flames at various Lewis numbers. Combust. Sci. Technol..

[80] Bray KNC, Moss JB (1977). A unified statistical model for the premixed turbulent flame. Acta Astronaut..

[81] Verma S, Lipatnikov AN (2016). Does sensitivity of measured scaling exponents for turbulent burning velocity to flame configuration prove lack of generality of notion of turbulent burning velocity?. Combust. Flame.

[82] Lipatnikov AN, Chomiak J (2002). Turbulent burning velocity and speed of developing, curved, and strained flames. Proc. Combust. Inst..

[83] Sabelnikov VA, Lipatnikov AN (2010). Rigorous derivation of an unclosed mean G-equation for statistically 1D premixed turbulent flames. Int. J. Spray Combust. Dynamics.

